# Invasion of midgut epithelial cells by a persistently transmitted virus is mediated by sugar transporter 6 in its insect vector

**DOI:** 10.1371/journal.ppat.1007201

**Published:** 2018-07-27

**Authors:** Faliang Qin, Wenwen Liu, Nan Wu, Lu Zhang, Zhongkai Zhang, Xueping Zhou, Xifeng Wang

**Affiliations:** 1 State Key Laboratory for Biology of Plant Diseases and Insect Pests, Institute of Plant Protection, Chinese Academy of Agricultural Sciences, Beijing, China; 2 Biotechnology and Germplasm Resources Institute, Yunnan Academy of Agricultural Sciences, Yunnan Key laboratory of Agricultural Biotechnology, Kunming, China; University of Cambridge, UNITED STATES

## Abstract

Insect transmission is obligatory for persistently transmitted viruses because the vector insect is the only means of virus spread in nature. The insect midgut is the first major barrier limiting virus acquisition, but the mechanisms by which viruses are able to cross the cell membrane and then infect the midgut epithelial cells of the insect have not been elucidated completely. Here, we found that the outer capsid or nucleocapsid protein (NP) of three viruses can interact and colocalize with sugar transporter 6 that is highly expressed in the midgut of *Laodelphax striatellus* (LsST6). In contrast, LsST6 did not interact with the NP of rice grassy stunt virus, which cannot be transmitted by the same planthopper. LsST6 not only altered the cellular location of viral proteins and then colocalized with them in the cell membrane, but also mediated the entry of rice stripe virus (RSV) particles into *Spodoptera frugiperda* 9 (Sf9) cells that expressed the heterologous gene *LsST6*. We further showed that RSV particles initially bound to the cell membrane of midgut epithelial cells where it colocalized with LsST6, and then invaded the cytoplasm. When LsST6 expression was knocked down, viral titre, acquisition percentage and transmission efficiency of the treated insect decreased significantly, but virus replication was not affected. This work thus uncovered a strategy by which LsST6 mediates viral entry into midgut epithelial cells and leads to successful transmission by the insect vector.

## Author summary

Sap/blood-feeding arthropods are major vectors of viruses that infect plants and vertebrates. Studies on the insect midgut, the first barrier for virus transmission, and its interactions with viruses and parasites are fundamental to understanding the transmission mechanism in vector insects and the epidemics caused by the vectored pathogen. Some putative receptors in arthropods have been discovered by *in vitro* protein interactions, but *in vivo* evidence is still lacking. Here, we found that the specific interaction between viral nucleocapsid protein and vector sugar transporter 6 of *Laodelphax striatellus* (LsST6) determines whether the virus can invade midgut epithelial cells or not. These results provide direct evidence that LsST6 is an essential and key factor in crossing the midgut infection barrier for viruses, especially for RSV. This vector protein may be a promising target for blocking transmission of diverse plant viruses. Our discovery has important implications for better understanding the interaction among host–virus–insect vector and disease epidemics caused by plant and animal arboviruses.

## Introduction

Many viruses persistently transmitted by arthropods cause serious diseases in plants, animals and humans. More than 76% of plant viruses and 40% of mammalian viruses are transmitted to the hosts by specific arthropods, mainly planthoppers, aphids, mosquitoes, and ticks [1, 2]. Frequent epidemics of viral diseases in rice, wheat and vegetables are largely attributed to high populations and viral transmission efficiency of the insect vectors [3–6]. Similarly, viruses that cause diseases in humans and animals such as dengue fever, Zika fever and Japanese encephalitis, are vectored by different species of *Aedes* mosquitoes and are endemic in many areas of the developing world [7–10]. Understanding the virus–insect vector interaction and transmission mechanisms will provide important information on the epidemics of the diseases caused by plant and animal arboviruses and lead to the development of better control strategies.

Plant viruses transmitted in a persistent propagative manner and animal arboviruses follow a similar circulative route within their insect vectors. After they are acquired from plant sap or blood ingested by the insect, the virions must first cross the cell membrane of the midgut epithelial cells where the viral particles multiply [11]. They must then leave the midgut to disseminate to other tissues including the salivary glands, from where they can be transmitted to new hosts [12]. During the circulative process, arboviruses must overcome multiple barriers, including the infection and dissemination barriers of the midgut, salivary gland escape barrier, and transovarial barrier [13, 14].

Previous studies showed that *Aedes aegypti* cannot be infected by eastern equine encephalomyelitis virus after ingesting viruliferous blood; however, it can transmit this virus after a virus suspension is directly injected into the insect’s abdomen [15]. Many plant viruses can also be transmitted by an insect that is not a natural host after the virus is injected into the hemocoel of the insect [16, 17], thus bypassing the midgut infection barrier, the first major barrier that viruses encounter and an important factor limiting virus transmission [1, 18, 19]. To overcome the midgut barrier, viruses have evolved different strategies. The entry of rice dwarf virus into cultured cells of its vector insect and of tomato yellow leaf curl virus midgut in its vector *Bemisia tabaci*, is mediated by clathrin-dependent endocytosis [20, 21], whereas southern rice black-streaked dwarf virus (SRBSDV) induces the formation of tubules as a vehicle for viral spread in infected epithelial cells of *Sogatella furcifera* [22].

The small brown planthopper, *Laodelphax striatellus* (Hemiptera: Delphacidae), is an important vector because it transmits numerous viruses that cause serious diseases of staple crops such as rice stripe virus (RSV), rice black-streaked dwarf virus, maize rough dwarf virus, northern cereal mosaic virus and barley yellow striate mosaic virus [4, 6, 23]. These plant viruses infect and replicate in *L*. *striatellus* and are retained by the vector insect throughout their life, as are vertebrate-infecting arboviruses [24–26]. In most experiments when feeding on RSV-infected plants, less than 30% of the insects acquire the virus [27–29]. A high affinity line of *L*. *striatellus* attained an acquisition level of about 50–60% after 4 days of acquisition feeding on RSV-infected rice plants, but four other lines reached a level of less than 10% after 8–11 days of acquisition feeding [30]. However, once acquired, the virus will replicate and be transmitted by vector insects at a moderate to very high rate [24, 31]. Although various biotic and abiotic factors affect virus acquisition by the vector insect, the epithelium, intercellular junctions, and basal lamina of the midgut present further barriers to viral entry and dissemination [13, 32].

The genome of RSV consists of four single-stranded RNAs (RNA1-4), which can encode at least seven proteins including the major nucleocapsid protein (NP) encoded by the ORF at the 5′ half of the viral complementary RNA3 [33]. NP is considered the key viral component for specifically interacting with the vector components and may play an important role in persistent transmission process. In previous studies, 66 proteins (including LsST6) were identified as being able to interact with the NP of RSV. Among these proteins, we chose several proteins according to molecular function and biological pathway to further investigate their function in virus transmission. CPR1 was demonstrated to stabilize the viral particles in the hemolymph [34], while vitellogenin, the precursor of a yolk protein in the insect, mediates virus entry into the ovary [35]. However, the proteins involved in the ability of RSV to overcome the midgut infection barrier were not identified. Because the sugar transporter Glut1 of human acts as a receptor for human T cell leukemia virus (HTLV) infection [36], and because a membrane protein named sugar transporter 6 of *L*. *striatellus* (LsST6) is highly expressed in the midgut, we selected LsST6 for further study. Our results showed it is an essential and key factor for RSV to cross the midgut infection barrier in vector insects.

## Results

### Analysis of the interaction between LsST6 and RSV NP or outer capsid protein of other viruses

The full-length *LsST6* (GenBank accession: MG589412), amplified from total RNA of *L*. *striatellus* using RT-PCR and 5’RACE, contained a 1470-bp open reading frame (ORF) encoding a predicted 489-amino-acid (aa) protein, which had 85.6% identity with sugar transporter NlST6 in the brown planthopper (*Nilaparvata lugens*) (S1 Fig). LsST6 belongs to the major facilitator superfamily (MFS) of membrane transport proteins because it has two symmetrical six-TMS (transmembrane spanner) units within a single polypeptide chain and a GRK domain conserved between TM2 and TM3 (S2 Fig).

**Fig 1:**
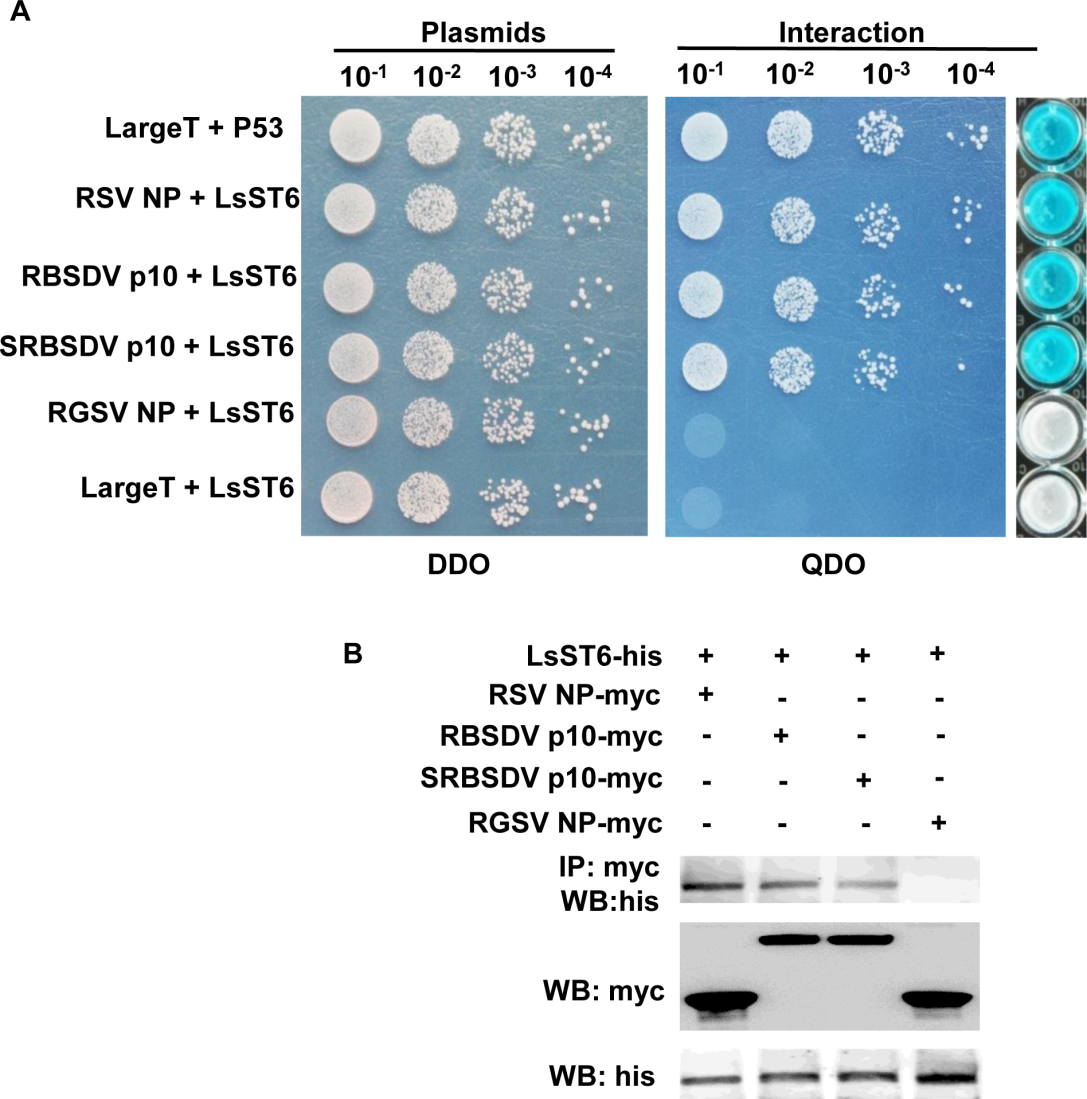
LsST6 interacted with viral proteins *in vivo* and *in vitro*. (A) Interaction between LsST6 and the outer capsid or nucleocapsid protein of different viruses detected using an *in vivo* yeast two-hybrid assay. Yeast strain NMY51 was cotransformed with LsST6 and the respective viral proteins. After 10^−1^ to 10^−4^ times dilution, the yeast cells were plated onto DDO (SD-trp-leu) and QDO (SD-trp-leu-his-ade-20 mM 3-AT) medium plates, respectively. Clones grown on DDO were selected for β-galactosidase activity assay. Large T + P53 was used as the positive control; Large T + LsST6 served as the negative control. (B) Confirmation of the interaction by *in vitro* coimmunoprecipitation (Co-IP). Sf9 cells were cotransfected with the respective recombinant bacmids (LsST6 fused with His-tag and viral proteins linked with Myc-tag) for protein expression. After 72 h, cells were harvested, lysed in lysis buffer, then incubated with anti-Myc antibody and protein A/G plus agarose beads for IP. The anti-Myc antibody or anti-His antibody was used for western blots.

A yeast two-hybrid (Y2H) assay based on split ubiquitin was used to verify whether LsST6 interacts *in vivo* with the NP of RSV and rice grassy stunt virus (RGSV) or the p10 of RBSDV and SRBSDV. In nature, *L*. *striatellus* can acquire RSV, RBSDV and SRBSDV, but not RGSV. The Y2H results showed that the yeast cells cotransformed with LsST6 and RSV NP, RBSDV p10 or SRBSDV p10, respectively, grew on the selective plates, but those with RGSV NP did not (Fig 1A). A similar result was obtained in a β-galactosidase activity assay (Fig 1A). Further, we exploited the cells of *Spodoptera frugiperda* 9 (Sf9) to coexpress LsST6 and the respective viral proteins for *in vitro* coimmunoprecipitation (Co-IP). LsST6 was labeled with a His tag and viral proteins with Myc tags at their C termini. The result showed that the anti-Myc antibody coimmunoprecipitated LsST6 that was coexpressed with RSV NP, RBSDV p10 or SRBSDV p10 in Sf9 cells, but not with RGSV NP (Fig 1B). All these results demonstrated that LsST6 only interacted with the proteins encoded by viruses that can be acquired by *L*. *striatellus*.

### LsST6 altered the cellular location of the interacting viral proteins in Sf9 cells

**Fig 2:**
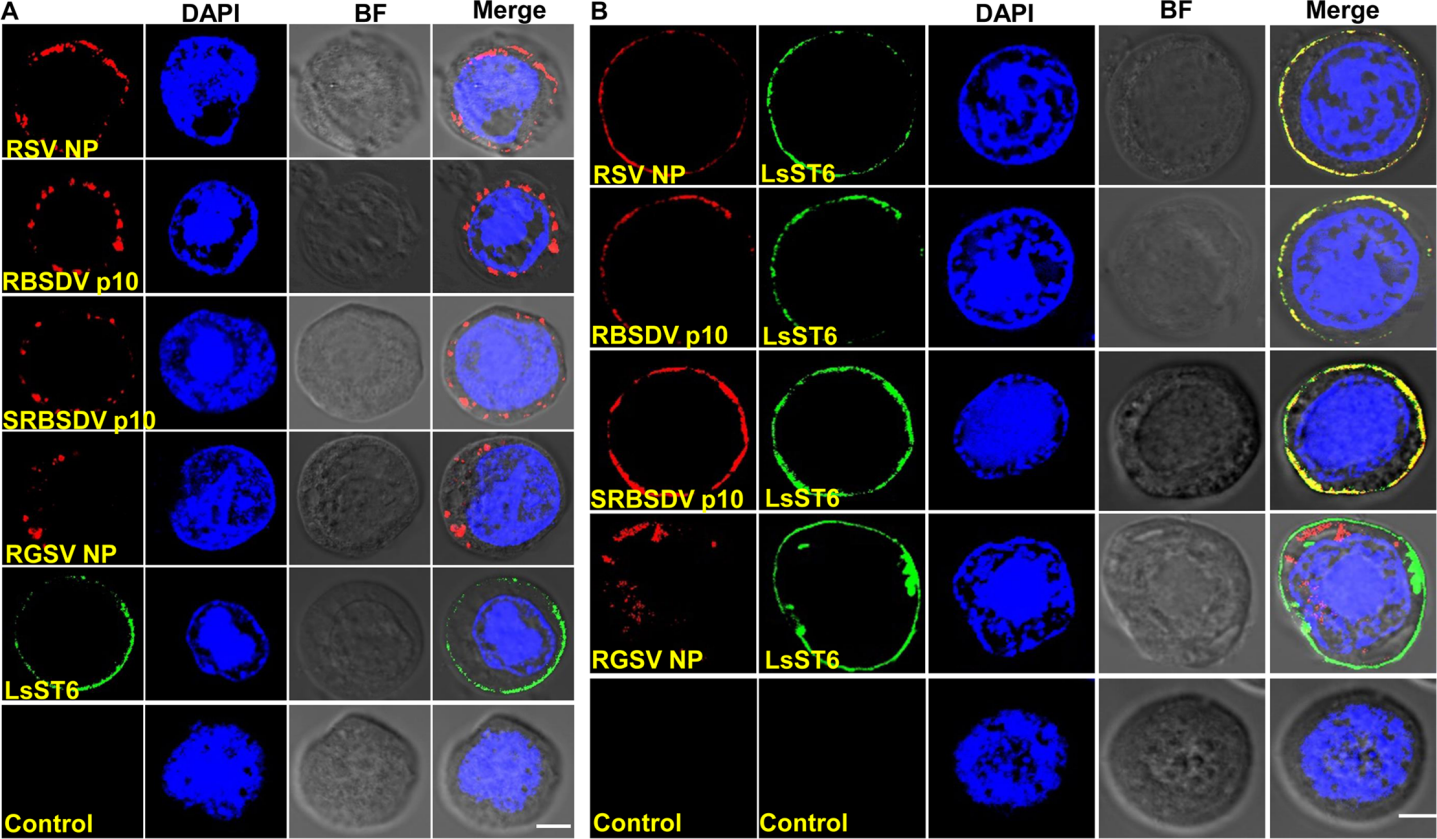
LsST6 affected the localization of interacting viral proteins in Sf9 cells. (A) Localization of LsST6 and outer capsid or nucleocapsid protein of different viruses in Sf9 cells. The recombinant bacmids (LsST6 and viral proteins) were individually used to transfect Sf9 cells, then observed with LSCM. (B) Colocalization of LsST6 and outer capsid or nucleocapsid protein of different viruses in Sf9 cells. LsST6 and the respective viral proteins were coexpressed in Sf9 cells and observed with LSCM 72 h after transfection. LsST6 was labeled with Dylight 488 (green); viral proteins were labeled with Dylight 549 (red). Cell nucleus was stained with DAPI (blue). Noninfected Sf9 cells served as the negative control. Scale bars, 10 μm.

Sf9 cells were transfected using a recombinant baculovirus that produces LsST6 or different viral proteins. Laser scanning confocal microscopy (LSCM) revealed that LsST6 (green) was mainly localized in the cell membrane, whereas viral proteins (red) localized in the cytoplasm of Sf9 cells (Fig 2A). Interestingly, RSV NP and p10 of RBSDV and SRBSDV moved from the cytoplasm to the cell membrane and colocalized with LsST6, while RGSV NP remained in the cytoplasm, separated from LsST6 when Sf9 cells coexpressed the respective viral protein and LsST6 (Fig 2B). The result suggested that the expression of LsST6 only changed the position of each interacting viral protein in Sf9 cells, and then colocalized with the protein on the cell membrane of Sf9 cells.

### LsST6 expression in different tissues and localization in the midgut epithelium of *L*. *striatellus*

**Fig 3:**
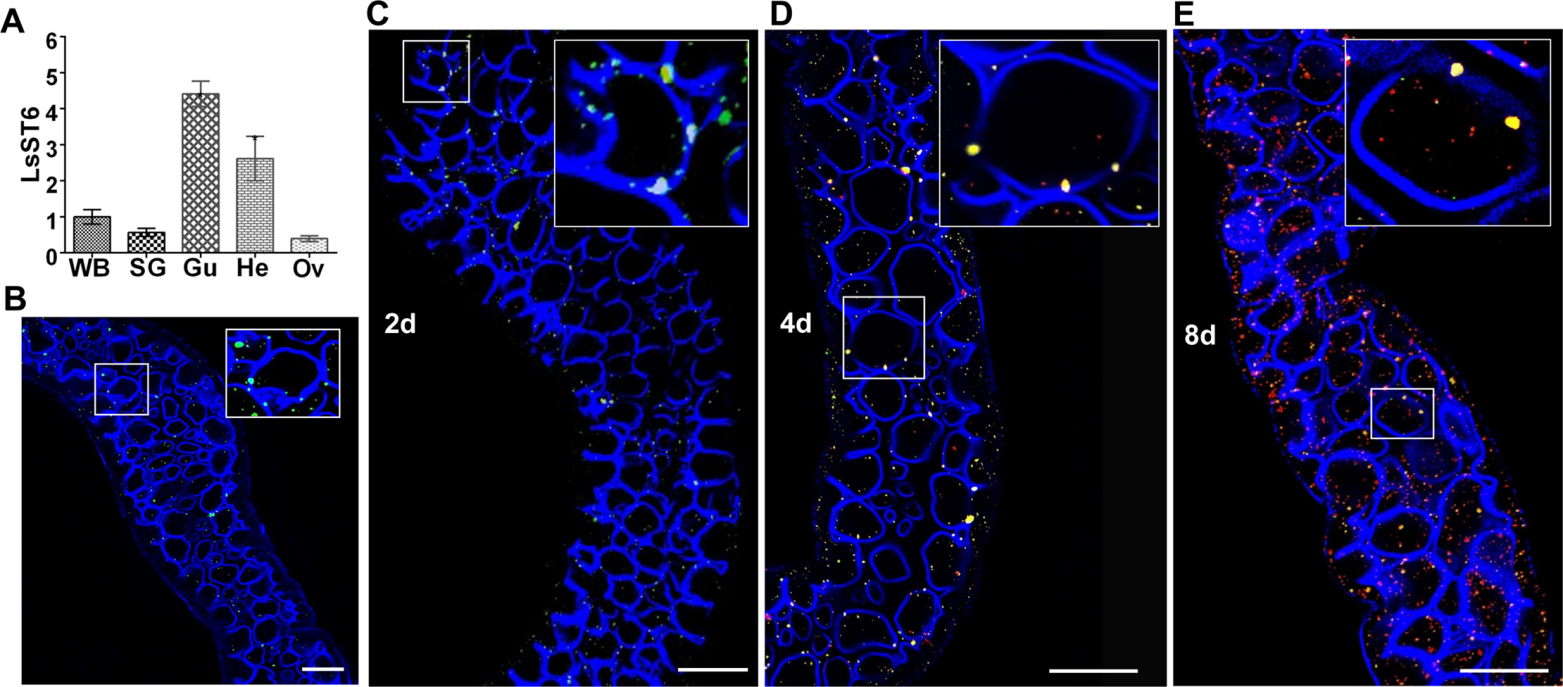
LsST6 is involved in rice stripe virus invasion of the midgut epithelial cells of *L*. *striatellus*. (A) LsST6 expression in different tissues. Total RNA was extracted from the salivary gland, gut, hemolymph and ovary for assay by RT-qPCR. The whole insect body served as the control. Means ± SEM of three independent experiments are shown (*P* < 0.01; one-way ANOVA, least significant difference [LSD] test). WB: whole body; SG: salivary glands; Gu: gut; He: hemolymph; Ov: ovary. (B) Localization of LsST6 in midgut epithelial cells. Excised midgut was incubated with anti-LsST6 antibody labeled with Dylight 488 (green) and observed with LSCM. Dylight 633 phalloidin was used to label actin (blue) of midgut epithelial cells. Scale bars, 50 μm. (C-E) Colocalization of LsST6 and RSV on membrane of midgut epithelial cell during RSV invasion. Excised midguts were incubated with anti-LsST6 labeled with Dylight 488 (green) and anti-RSV labeled with Dylight 549 (red) at 2 days (C), 4 days (D) and 8 days (E) after a 2-day access acquisition period (AAP), then observed with CLSM. Scale bars, 50 μm.

The salivary gland, gut, hemolymph and ovary were excised separately from adult insects, then total RNA was extracted from individual tissues to quantify LsST6 by RT-qPCR. The results showed that LsST6 had the highest expression in gut tissues, followed by the hemolymph, salivary glands and ovary (Fig 3A). The alimentary canal of the planthopper, which mainly comprises the esophagus (es), anterior diverticulum (ad), midgut (mg), hindgut (hg) and malpighian tubules (mt) (S3A Fig), was excised, and then incubated with anti-LsST6 antibody labeled with Dylight 488 (green). The confocal image showed that LsST6 was located in the cell membrane and cytoplasm of the midgut epithelial cells (Fig 3B).

**Fig 4:**
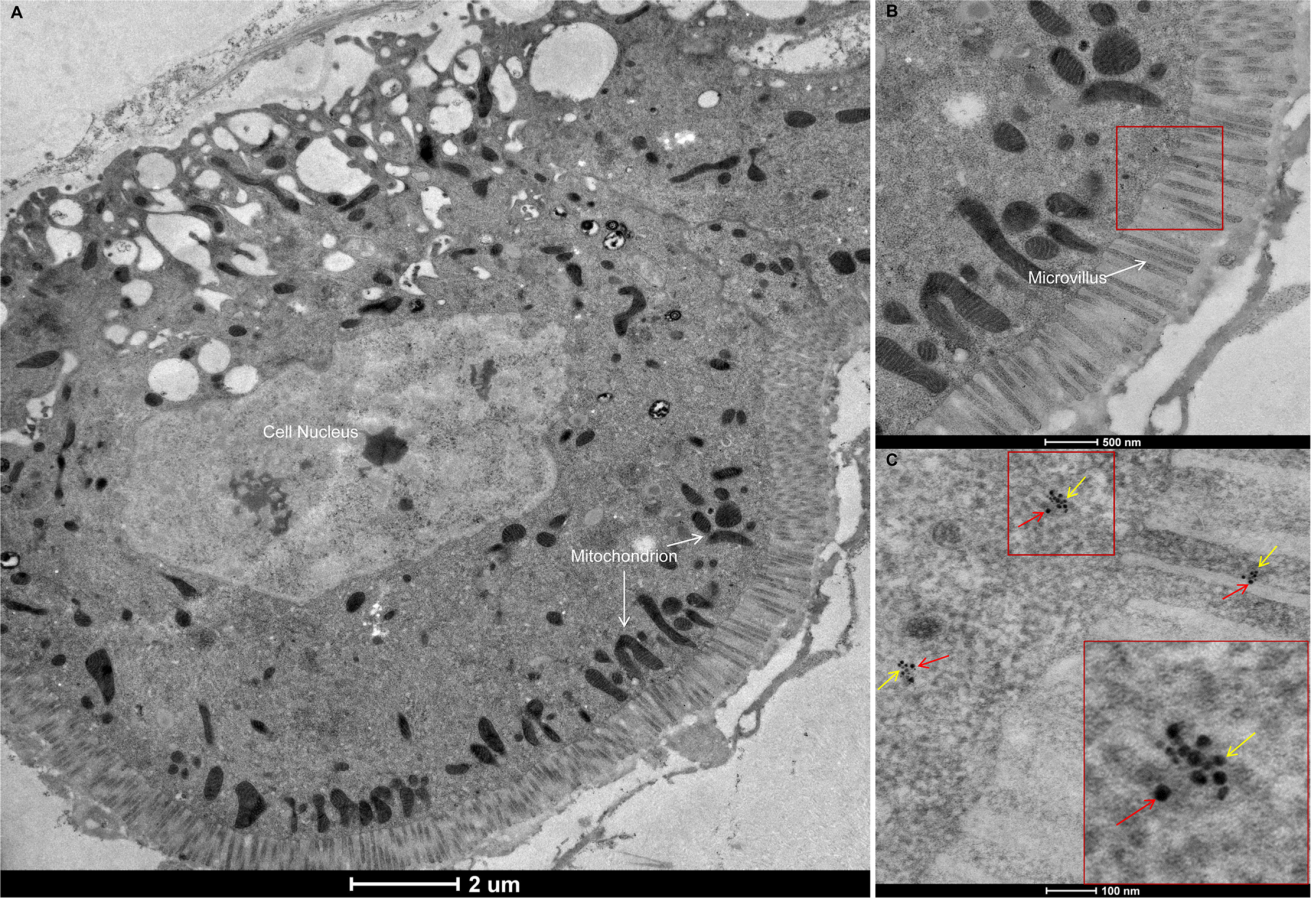
Immunoelectron microscopy to show colocalization of RSV particles and LsST6 in epithelial cells of *L*. *striatellus* allowed to feed for 48 h on RSV-infected rice plants. (A) Immunoelectron micrograph of midgut epithelial cell. (B, C) Colocalization of RSV particle and LsST6 in microvilli and cytoplasm. Red box: Magnified area. Red arrows: 10-nm gold-conjugated goat anti-rabbit IgG against RSV used to detect virus; yellow arrows: 5-nm gold-conjugated goat anti-mouse IgG against LsST6.

The distribution of RSV particles in the alimentary canal over time was also visualized by LSCM using anti-RSV antibody labeled with Dylight 549 (red). At 2 d after a 2-d acquisition access period (AAP), a few RSV particles were observed in several epithelial cells of the midgut. By 4 d after the AAP, RSV particles had replicated and spread to the neighboring epithelial cells, and by 8 d after the AAP, RSV particles were observed in the entire alimentary canal (S3B Fig). Thus, the original RSV infection site was the epithelium of the midgut. Interestingly, at 2 d after the AAP, LsST6 had colocalized with RSV in the cell membrane of epithelial cells (Fig 3C). At 4 d and 8 d after the AAP, viral particles were still colocalized with LsST6 in the cell membrane of the epithelial cells, and some particles had already invaded the cytoplasm, but the fluorescent signals indicative of the viral titre were stronger at 8 d (Fig 3D and 3E). When we used immunoelectron microscopy to examine the virus-infected midgut epithelium, RSV particles also colocalized with LsST6 on the microvilli of cell membranes and cytoplasm (Fig 4). This evidence strongly suggested that LsST6 is a key factor for enabling RSV entry into the midgut epithelium of the planthopper.

### LsST6 mediated the entry of RSV particles into Sf9 cells

**Fig 5:**
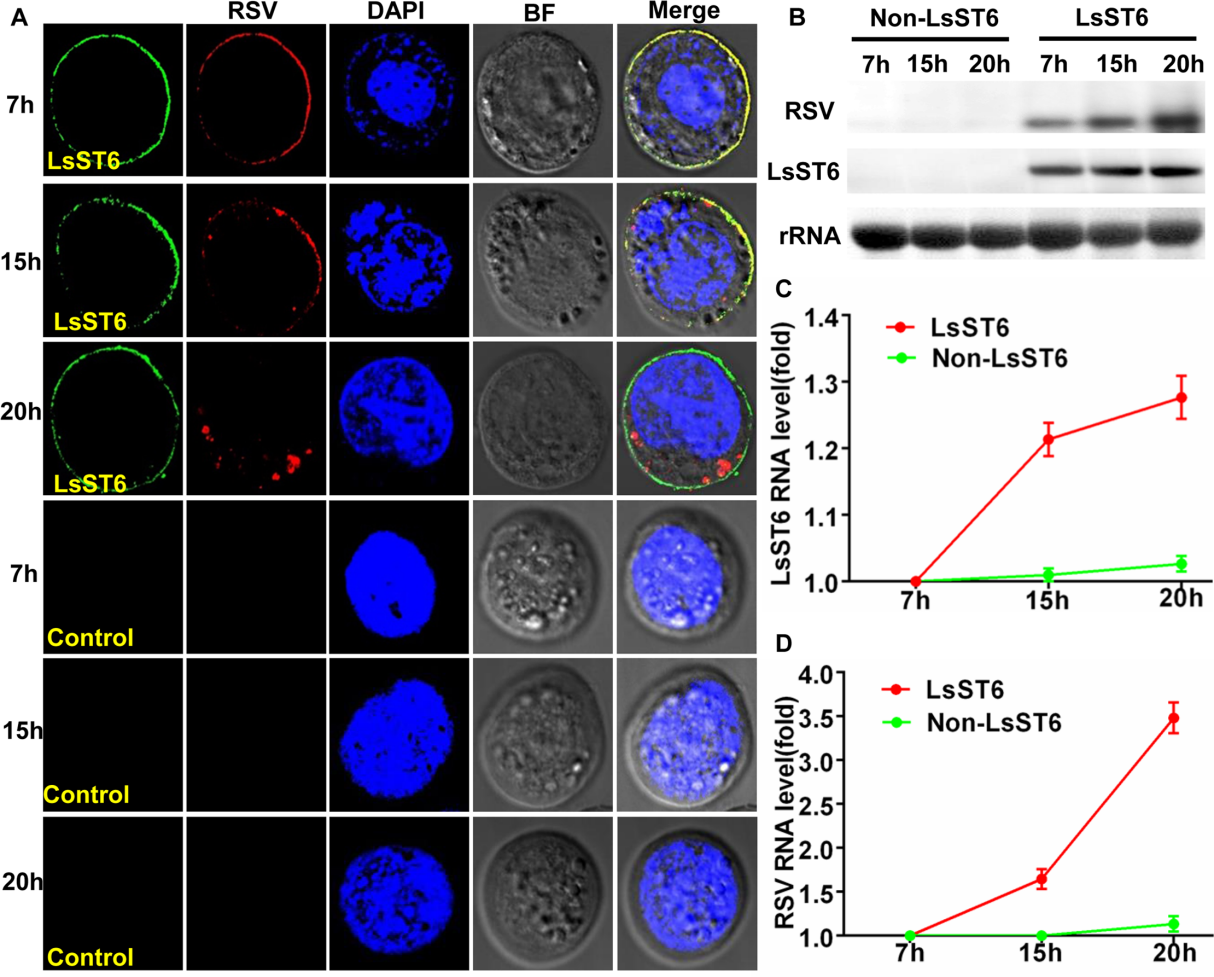
RSV particles successfully invaded Sf9 cells that expressed the heterologous gene *LsST6*. (A) RSV particle invasion of Sf9 cells was mediated by binding with LsST6. RSV particles were added to Sf9 cells, which had been transfected by recombinant bacmid plasmid of LsST6 at 48 h. After 7 h, 15 h and 20 h incubation with RSV particles, Sf9 cells were treated with anti-LsST6 labeled with Dylight 488 (green) and anti-RSV labeled with Dylight 549 (red) and observed with LSCM. Scale bars, 10 μm. (B-D) The accumulation level of RSV particles increased when LsST6 was expressed in Sf9 cells. Total RNA was extracted from Sf9 cells at 7, 15 and 20 h after adding virus particles and then probed by DIG-northern blot using specific probes (B) and by RT-qPCR (C and D) to analyse the levels of LsST6 and RSV RNA. The mRNA levels of the housekeeping gene ECD were used as an internal control, and mRNA for LsST6, and RNA for RSV level of LsST6 and RSV at 7 h was set to 1. (B–D) Mean ± SEM of three independent experiments (*P* < 0.01, one-way ANOVA, LSD test).

**Fig 6:**
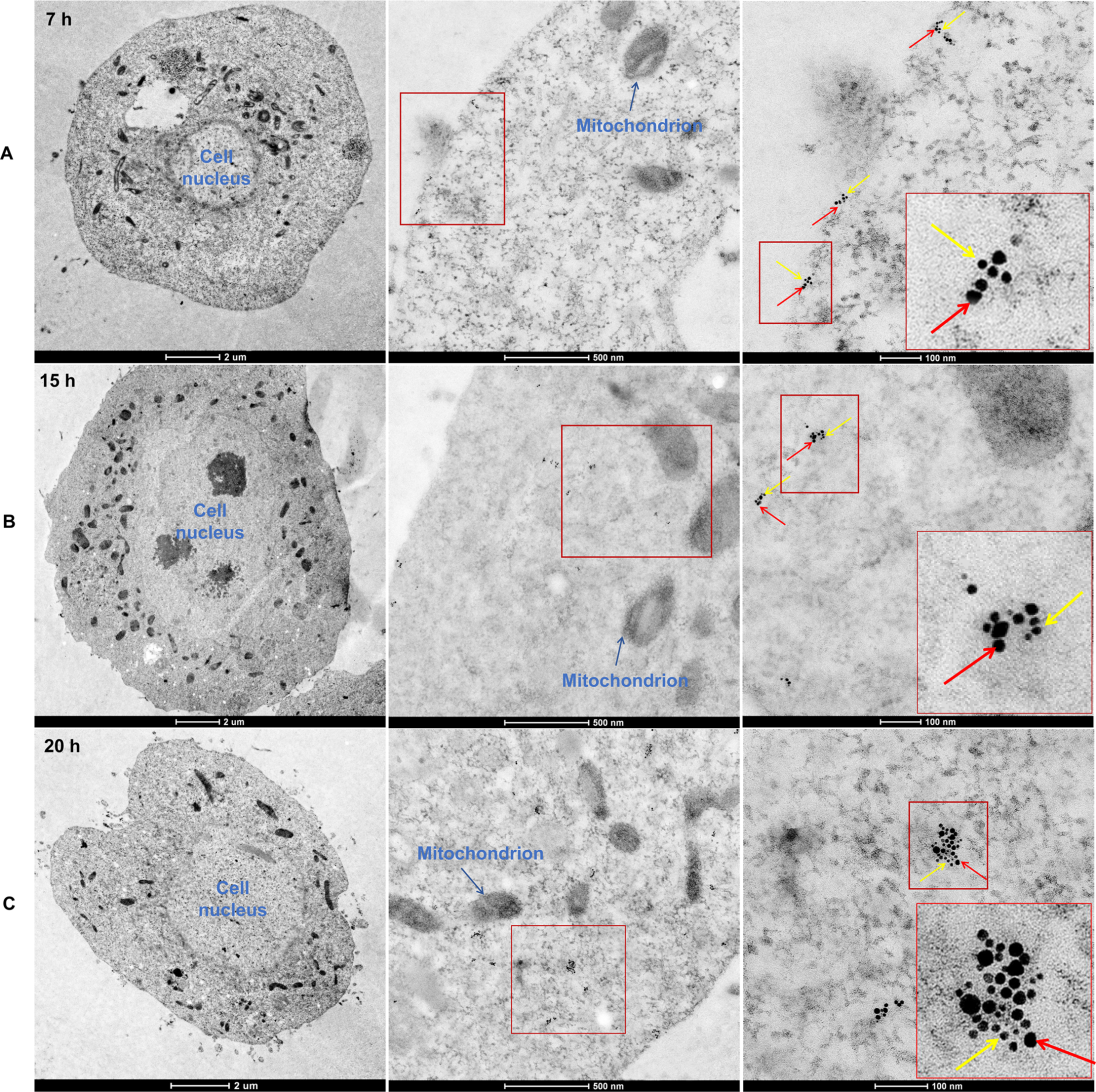
Immunoelectron microscopy showing RSV particle entered Sf9 cells. (A) RSV particles and LsST6 colocalized in the cell membrane by 7 h. (B) RSV particles had entered the cytoplasm. (C) Numerous RSV particles were distributed in the cytoplasm. Red arrows: 10-nm gold-conjugated goat anti-rabbit IgG against RSV used to detect virus; yellow arrows: 5-nm gold-conjugated goat anti-mouse IgG against LsST6.

RSV particles were added to a liquid culture of Sf9 cells that expressed the heterologous gene *LsST6*; by 7 h, they had colocalized with LsST6 on the cell membrane of Sf9 cells. Few viral fluorescence signals were found in the cytoplasm by 15 h, but more signals were detected in the cytoplasm by 20 h (Fig 5A). Immunoelectron microscopy also consistently showed the same results; RSV particles colocalized with LsST6 in the cell membrane at 7 h (Fig 6A) and were observed in the cytoplasm of Sf9 cells at 15 h and 20 h (Fig 6B and 6C).

We then quantified the mRNA level for RSV and LsST6 at various times using northern blots and RT-qPCR. The RSV mRNA level was obviously higher at 20 h than at 7 h when LsST6 was expressed (Fig 5B). The RT-qPCR data also supported the northern blot results: at 15 h and 20 h, the level of viral RNA was 1.5 and 3.5 times higher, respectively, than at 7 h (Fig 5D), whereas the level of LsST6 mRNA was approximately 1.2 and 1.3 times higher, respectively, at 15 h and 20 h (Fig 5C). That viral particles passed though the cell membrane, mediated by LsST6, revealed that LsST6 played a critical role in RSV entry Sf9 cells.

### Inhibiting the expression of *LsST6* prevented RSV entry into midgut epithelial cells

**Fig 7:**
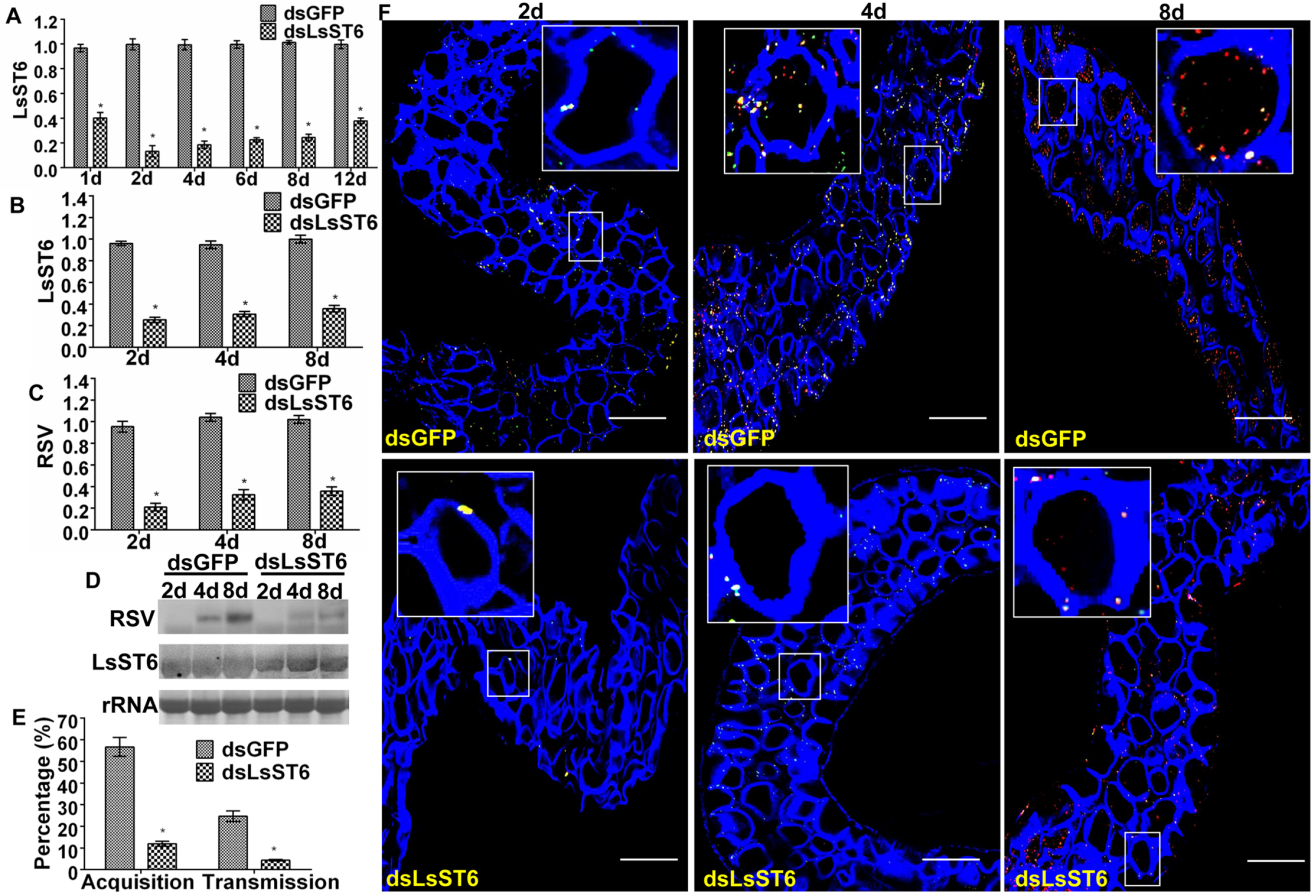
Knockdown of *LsST6* expression interfered with virus entry into midgut epithelial cells of healthy planthoppers. (A) RNA interference mediated by microinjection with dsLsST6. Third-instar and nonviruliferous nymphs were injected with dsLsST6 or dsGFP using an Auto-Nanoliter Injector, then fed on healthy rice seedling. *LsST6* mRNA expression level was quantified by RT-qPCR at different times. (B) *LsST6* RNA levels were reduced after injection with dsLsST6 as quantified by RT-qPCR. (C) RNA level of RSV quantified by RT-qPCR. (D) RNA levels of RSV and LsST6 were quantified by DIG-northern blot. Nonviruliferous SBPHs that had been injected with either dsLsST6 or dsGFP were fed on RSV-infected rice for a 2-day acquisition access period (AAP), then collected at 2, 4 and 8 days to quantify RNA. Level of the housekeeping gene *actin* was used as an internal control. (E) Percentage of RSV-positive insects and transmission efficency decreased significantly compared with the control that was injected with dsGFP. Total RNA was extracted from insects at 8 days after a 2-day AAP or from rice seedling at 21 days after a 10 h inoculation access period and used to detect RSV by RT-PCR using specific primers. (A–D) Mean ± SEM of three independent experiments (*P* < 0.01, one-way ANOVA, LSD test). (F) Localization of LsST6 and RSV in midgut epithelial cells after knockdown of *LsST6* expression. Excised midguts were incubated with anti-LsST6 (green) and anti-RSV (red) antibodies and observed with LSCM. Scale bars, 50 μm.

When dsRNA of LsST6 (dsLsST6) was injected into third-instar nymphs, LsST6 mRNA level in the midgut had declined by 61% and 87% at 1 d and 2 d compared with the control (insects injected with dsGFP). Subsequently, LsST6 mRNA level remained 20–40% lower than in the control group injected with dsGFP (Fig 7A). We then allowed third-instar nymphs that had been injected with dsLsST6 or dsGFP to feed on RSV-infected plants for a 2-d AAP, then quantified RSV and LsST6 mRNA levels in treated insects after different times using RT-qPCR and northern blots. The RSV mRNA level in insects injected with dsLsST6 was 22% to 35% of that in SBPH injected with dsGFP (Fig 7C), indicating significant interference with LsST6 expression (Fig 7B). The northern blot also showed that the quantity of RSV RNA was evidently lower than in the group injected with dsGFP after 4 and 8 d (Fig 7D). In addition, viral acquisition and transmission by dsLsST6-injected SBPHs decreased by nearly 80% compared with those injected with dsGFP (Fig 7E, S3 Table). Confocal images also showed fewer RSV infection sites and fewer RSV particles in a single epithelial cell than in the control at 2 and 4 d (Fig 7F). Overall, these results demonstrated that RSV initial infection of the midgut epithelial cells was inhibited when *LsST6* expression was knocked down.

### Inhibiting the expression of *LsST6* had no significant effect on RSV replication

**Fig 8:**
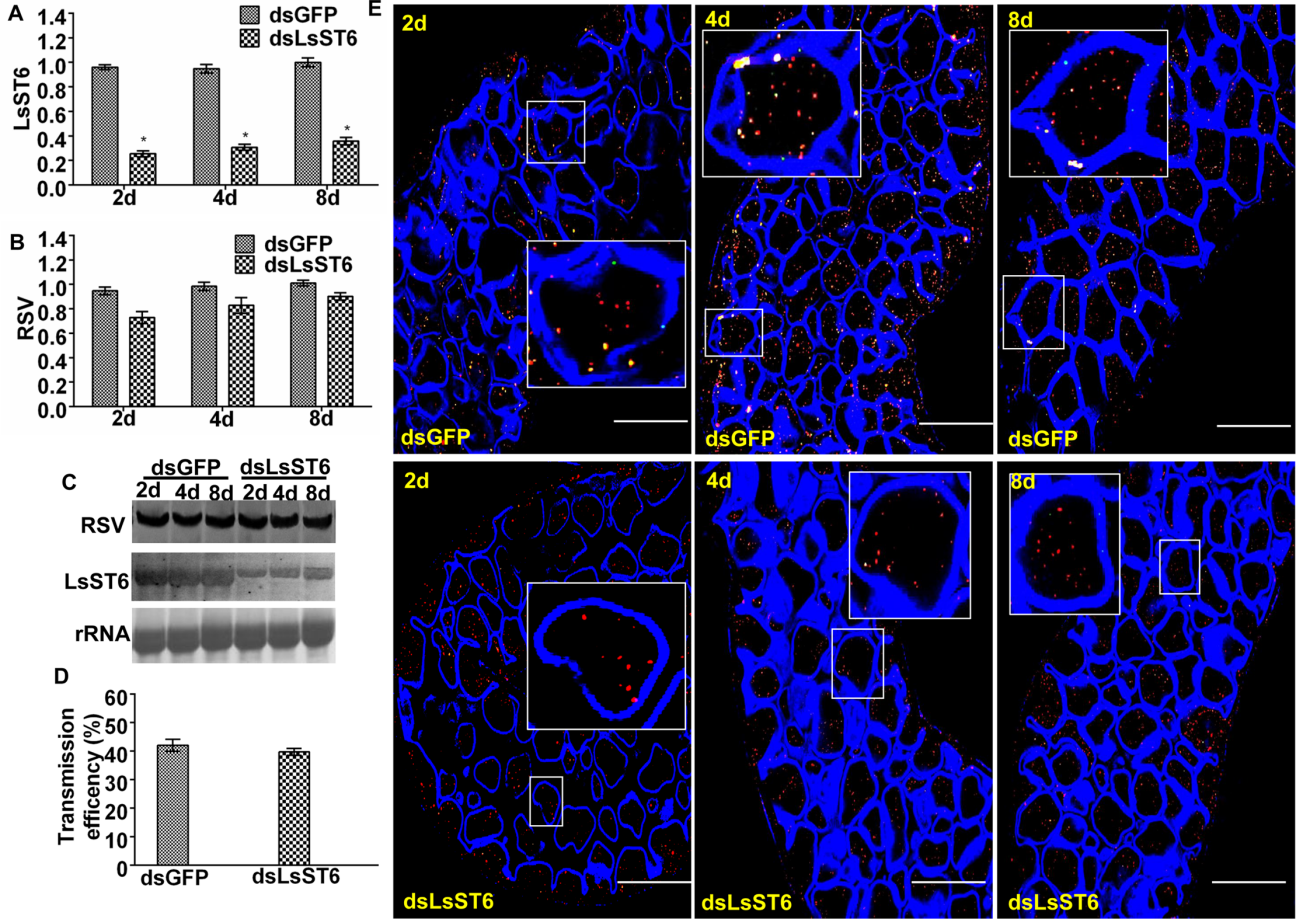
RNAi knockdown of *LsST6* had no effect on virus titre and transmission efficiency in viruliferous planthoppers. (A) *LsST6* RNA expression was inhibited by injection with dsLsST6. Viruliferous insects were fed on health rice seedlings and collected at 2, 4 and 8 days after the dsRNA injection, then RNA levels quantified by RT-qPCR. (B-C) RSV titre in viruliferous insects after injection. RSV RNA levels were quantified by (B) RT-qPCR and (C) DIG-northern blots. (D) Transmission efficiency of virus to rice seedlings by viruliferous insects after injection with dsLsST6. (A-D) Mean ± SEM of three independent experiments (*P* < 0.01, one-way ANOVA, LSD test). (E) Localization of LsST6 and RSV in midgut epithelial cells after injection with dsLsST6. Excised midguts at 2, 4 and 8 days were incubated with anti-LsST6 (green) and anti-RSV (red) antibodies and observed with LSCM. Scale bars, 50 μm.

We also injected viruliferous insects with dsLsST6 or dsGFP. Compared with the RSV mRNA level in the insects injected with dsGFP, the level in the dsLsST6-injected insects only decreased by 10–20% at 2, 4 and 8 d after injection, while the *LsST6* mRNA level decreased by 65–70% (Fig 8A and 8B). The northern blot assay showed that the change in RSV mRNA levels followed a similar trend in insects after injection with dsLsST6 or dsGFP (Fig 8C). Confocal images also suggested that the quantity of RSV in epithelial cells was similar to the control (Fig 8E). In addition, two groups of treated viruliferous insects had a similar transmission efficiency (Fig 8D, S4 Table). Therefore, LsST6 has no significant effect on virus replication.

## Discussion

The insect midgut consists mainly of a single layer of epithelial cells, with extensive microvilli on the lumen side and a porous basal lamina on the hemocoel side [37, 38]. The midgut absorbs the nutrients necessary for insect survival and provides an environment for the development and multiplication of viruses and parasites [39, 40]. The midgut epithelial cells have been identified as the initial infection site and the first barrier to virus invasion [1, 13, 41]. Our results on the distribution of RSV particles in the alimentary canal over time also demonstrated that the midgut epithelial cells of *L*. *striatellus* served as the initial infection site of RSV. After successful invasion of the midgut, RSV began its replication process, then spread into neighboring cells.

Most viruses invade insect epithelial cells via specific interaction between the structural proteins of the virus and the cell surface receptor complexes in vectors, similar to their infection of host cells [18, 19, 42, 43]. Viral surface components have been well demonstrated to play an important role in virus infection and transmission [44, 45], and putative surface receptors including glycans and glycoconjugates for flaviruses have been found in host and insect cells [46, 47]. A 32-kDa laminin-binding protein and 35-kDa prohibitin that mediate entry of Venezuelan equine encephalitis virus and Dengue virus-2, respectively, into mosquito cells have been identified [48, 49]. Membrane alanyl aminopeptidase N has been identified in the pea aphid as responsible for the entry of the pea enation mosaic virus into the aphid gut [50]. Most of these putative receptors in insects were discovered by *in vitro* interactions; *in vivo* evidence is still lacking.

We thus used cellular and molecular biological techniques to advance our understanding of the interaction between virus and insect vector. Here, we found that LsST6 not only strongly interacts with RSV NP *in vitro* and *in vivo*, but also alters the cellular location of NP and then colocalizes with it in the cell membrane of Sf9 cells. Moreover, LsST6 mediates the entry of RSV particles into Sf9 cells that expressed the heterologous gene *LsST6*. In the vector insect body, RSV initially binds to the cell surface of midgut epithelial cells where it colocalizes with LsST6 in the cell membrane. When expression of *LsST6* was knocked down in healthy insects injected with dsLsST6, viral titre and acquisition subsequently decreased significantly. Therefore, LsST6 plays an important role in facilitating virus invasion in both Sf9 model cells and the midgut epithelial cells of the vector insect, *L*. *striatellus*.

Our previous bioinformatic analysis revealed that LsST6 has 85% similarity with NlST6, a facilitative glucose/fructose transporter in brown planthoppers (*N*. *lugen*) [51]. They both belong to the major facilitator superfamily (MFS) of transporters, which are ubiquitous among organisms and enable the import and export of essential nutrients and ions (not just sugars), the excretion of metabolic end products and deleterious substances and communication of the cells with the environment [52, 53]. Some members of the MFS are also exploited by viruses to invade host cells. Glut1, a receptor for HTLV [36], was recently found to mediate glucose transport, which regulates human immunodeficiency virus (HIV) infection in human T cell lines [54]. Glut1 of shrimp is thought to be a putative cell surface receptor for white spot syndrome virus [55]. Feline leukemia virus subgroup C receptor (FLVCR), another member of the MFS, is considered to be the cell surface receptor for feline leukemia virus [56, 57]. HTLV and HIV infection of host cells are all regulated by Glut1-mediated glucose metabolism, via an increase in *Glut1* expression and to a change in the conformation of the protein [53,54]. These studies thus provide a precedent for the involvement of another MFS member, LsST6, in virus invasion of midgut epithelial cells in *L*. *striatellus* using a similar transport mechanism, rather than receptor-/clathrin-dependent endocytosis or membrane fusion and/or actin-based tubular structures to overcome the cell barriers. Knock down of the expression of *LsST6* in healthy insects of *L*. *striatellus*, resulted in a decrease in virus acquisition after they fed on RSV-infected rice plants compared with the control insects with the functional gene. The viral titre in viruliferous insects that were similarly treated by injection with dsLsST6 had no significant changes. These results suggest that LsST6 mediates RSV entry into cells but is not involved in virus replication. On the basis of our results, we propose that LsST6 on the cell membrane of epithelial cells in the midgut mediates RSV invasion during facilitative transport of glucose/fructose from the phloem sap of rice plants across the cell membrane.

**Fig 9:**
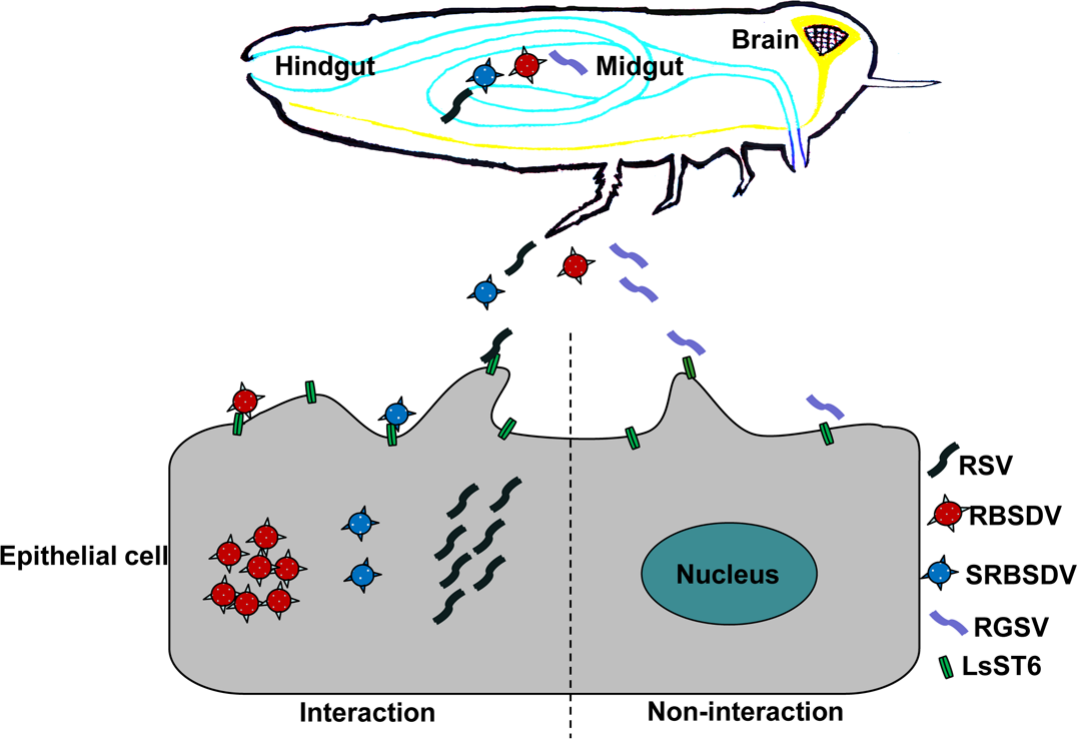
Model of virus entry into the midgut epithelial cell. The viruses (RSV, RBSDV, SRBSDV and RGSV) enter the lumen of the midgut with sap of virus-infected rice plants ingested by *L*. *striatellus*, then the virions bind to the cell membrane of midgut epithelial cells by specific interaction with LsST6. Only a compatible interaction results in the virions crossing the cell membrane to enter the epithelial cells, where they replicate and are disseminated to other body parts of the insect.

Interestingly, we found that LsST6 not only interacted with the outer capsid of RBSDV and SRBSDV, but also colocalized with each in the cell membrane of Sf9 cells, but it did not colocalize with NP of RGSV. Because *L*. *striatellus* can transmit both RSV and RBSDV but not RGSV, we consider that the two transmitted viruses may also require LsST6 to mediate their entry into midgut epithelial cells. Although the planthopper cannot transmit SRBSDV efficiently, previous evidence has shown that this virus does invade midgut tissues, but it does not spread into the hemolymph or other organs of SBPH [58, 59]. SRBSDV cannot break through the release barrier of the midgut because it cannot replicate enough to reach the threshold required for further spread, and/or the siRNA antiviral pathway has a direct role in controlling viral dissemination from the midgut [60, 61]. RGSV cannot invade the midgut epithelium of *L*. *striatellus*, because LsST6 did not interact or colocalize with the NP of RGSV. Therefore, LsST6 might specifically mediate initial infection by the numerous viruses that are transmitted by *L*. *striatellus*. Based on all the data obtained, we propose a model by which RSV overcomes the midgut infection barrier in vector planthopper. After entering the alimentary canal of the vector insect and arriving in the midgut, intact RSV particles can bind to the midgut epithelial cells, where the NP of RSV interacts specifically with sugar transporter 6 on the cell membrane and is transported into the epithelial cells, where it replicates and finally disseminates to other parts of the vector (Fig 9). This model should also be applicable to other viruses transmitted by *L*. *striatellus*.

In conclusion, our results provide direct evidence that LsST6 is essential for RSV invasion of the midgut epithelial cells in its insect vector. The fact that LsST6 can also interact and colocalize with the outer capsid or NP of other viruses transmitted by *L*. *striatellus* suggests that numerous arboviruses might use a similar vector protein to invade the midgut epithelium of the insect vector. This key vector protein could be used as a target for blocking virus transmission and lead to a new strategy to control outbreaks of diseases caused by arboviruses.

## Materials and methods

### Insects

Nonviruliferous and viruliferous *L*. *striatellus* with a high affinity for rice stripe virus were reared on healthy and RSV-infected rice seedlings (cv. Wuyujing 3), respectively [29, 34]. Every 3 months, the offspring of viruliferous insects were confirmed as RSV-positive using RT-PCR.

### Viruses

Viruses (RSV, RBSDV and SRBSDV)-infected rice leaves collected from the field were stored at −80°C in the lab (61) and RGSV-infected rice plant was provided by Prof. Taiyun Wei (Fujian Agriculture and Forestry University). RSV particles were extracted from RSV-infected rice leaves as described previously [62], and purified virions were stored at −80°C.

### Antibodies and reagents

The mouse monoclonal anti-RSV antibody was kindly provided by Prof. Jianxiang Wu (Zhejiang University). The rabbit anti-RSV antibody was the kind gift of Yan Huo (Chinese Academy of Sciences). An anti-LsST6 monoclonal antibody against the LsST6 peptide SKGDHNTEAALP was produced by Abmart (Shanghai, China). The following antibodies were obtained from the sources indicated: mouse monoclonal anti-Myc tag (cat. 66004, Proteintech), rabbit polyclonal anti-His tag (cat. 2365, Cell Signaling Technology), Dylight 488 goat anti-rabbit IgG (cat. A23220, Abbkine), Dylight 488 goat anti-mouse IgG (cat. A23210, Abbkine), Dylight 549 goat-anti-mouse IgG (cat. A23310, Abbkine), goat anti-mouse IgG+HRP (cat. 32430, Thermo), goat anti-rabbit IgG+HRP (cat. 32460, Thermo). Alexa Fluor 633 phalloidin was obtained from Invitrogen and 4’,6-diamidino-2-phenylindole (DAPI) was purchased from Sigma.

### Recombinant DNA constructs

The genes including RSV *NP*, RBSDV *p10*, SRBSDV *p10*, RGSV *NP* and *LsST6* were amplified using specific primers (S1–S2 Tables) and were subsequently cloned into the bait plasmid pDHB1 or prey plasmid pPR3-N (Dualsystems Biotech) to generate pDHB1-RSV NP, RBSDV p10, SRBSDV p10 or RGSV NP or pPR3-LsST6. Genes including RSV *NP*, RBSDV *p10*, SRBSDV *p10* and RGSV *NP* linked with a Myc tag sequence were cloned into plasmid pFastBac (Invitrogen), while *LsST6* was inserted into the BamHI/XbalI sites of pFastBacHTB (Invitrogen) vector containing a 6×His tag.

### Sf9 cell line

Sf9 cells were incubated in Sf-900 III SFM Serum Free medium containing 5% newborn calf serum at 27°C.

### Yeast two-hybrid assay

To confirm any interaction between LsST6 and RSV NP, RBSDV p10, SRBSDV p10 or RGSV NP, we used the DUALhunter starter kit, a yeast two-hybrid system based on the reconstitution of ubiquitin. A clone of yeast strain NMY51 was selected and incubated in 25 ml yeast peptone dextrose adenine agar (YPDA) at 30°C with shaking until the culture reached an OD_546_ of 0.6–0.8. The culture was collected by centrifugation, and the pellet was suspended in 1.5 ml water. Then, 1.5 μg bait vector plasmid (pDHB1-NPs/-p10s) and 1.5 μg prey vector plasmid (pPR3-LsST6) were added to 100 μl culture resuspended in 300 μl PEG/ lithium acetate Master Mix (50% PEG, 1 M lithium acetate and 125 μl single-stranded carrier DNA) and incubated in a 42°C water bath for 45 min. Finally, the mixture was collected by centrifugation at 700 × *g* for 5 min, and the pellet resuspended in 100 μl 0.9% NaCl (wt/vol), then plated onto selection plates of DDO (SD-trp-leu) and QDO (SD-trp-leu-his-ade) medium with 20 mM 3-aminotriazole (3-AT) and incubated for 3–4 days at 30°C. For distinguishing false-positive interactions, the clone grown on DDO was incubated on 1 ml liquid DDO overnight at 30°C with shaking until the culture reached an OD_546_ of 0.5–0.8 to check for β-galactosidase activity. The culture was then collected by centrifugation, and the pellet was added to 100 μl of freshly prepared lysis mixture (9.95 ml of one-step lysis and assay reagent with 50 μl of dye stock solution) provided in the HTX High-throughput β-galactosidase kit with a brief vortex.

### Cell transfection

Sf9 cells were transfected using Cellfectin II according to the manufacturer’s instructions. Briefly, 2 × 10^6^ cells were added to each well of a 6-well culture dish for at least 30 min. Then 2 μg recombinant bacmid–plasmid DNA encoding LsST6, RSV NP, RBSDV p10, SRBSDV p10 or RGSV NP was mixed with 8 μl Cellfectin II and incubated for 20 min. Then each mixture was added to a well of the 6-well culture dish and incubated at 27°C for 5 h. The transfection mixture was then removed and replaced with growth medium. Transient expression was measured 72 h later using LSCM and western blot.

### Coimmunoprecipitation (Co-IP) assay

Genes including RSV *NP*, RBSDV *p10*, SRBSDV *p10* and RGSV *NP* linked with a Myc tag sequence were cloned into plasmid pFastBac (Invitrogen), while *LsST6* was inserted into the BamHI/XbalI sites of pFastBacHTB (Invitrogen) vector containing a 6×His tag. Recombinant *LsST6* DNA was used to cotransfect Sf9 cells with recombinant RSV *NP*, RBSDV *p10*, SRBSDV *p10* or RGSV *NP* DNA during a 72-h incubation at 27°C, then cultures were collected and lysed in the lysis buffer (20 mM Tris-HCl pH 7.6, 150 mM NaCl, 0.5% NP-40, 5 mM EDTA, and complete protease inhibitor cocktail tablets) for 1 h on ice. After the cultures were centrifuged at 10,000 × *g* for 20 min at 4°C, 50 μl protein A/G plus agarose beads was added to the supernatant for 1 h at 4°C to decrease any nonspecific binding of proteins. Then the supernatant was incubated with 2 μl (1 μg/μl) anti-Myc antibody for 1 h, followed by incubation with protein A/G plus agarose beads at 4°C with end-over-end agitation for overnight. The protein A/G plus agarose beads were washed with washing buffer (20 mM Tris-HCl pH 7.6, 150 mM NaCl, 5 mM EDTA) 5 times, then mixed with 1× loading buffer (0.08 M Tris pH 6.8, 2.0% SDS, 10% glycerol, 0.1 M dithiothreitol, and 0.2% bromophenol blue) and finally boiled for 5 min. The cell lysates and IP cultures were separated on SDS-PAGE gels and transferred to nitrocellulose membranes. Membranes were incubated with antibody of anti-His or anti-Myc (1:3000) for 1.5 h at room temperature, then incubated with the secondary antibody-alkaline phosphatase-conjugated goat anti-mouse or rabbit IgG (1:5000) at 37°C for 1 h. Membranes were then incubated with a chemiluminescent substrate mixture and imaged using the imageQuant LAS 4000 mini biomolecular imager (GE Healthcare Life Sciences, USA).

### RSV particles infection of Sf9 cells

RSV particles (1.5 μg/μl) were added to the Sf9 cells transfected by recombinant bacmids LsST6 or empty bacmids at 48 h, then the sf9 cells were rinsed with PBS buffer 3 times and collected at 7, 15 and 20 h after RSV particles were added to the culture medium. To avoid the influence of residual inoculum, the cells are collected by centrifugation to remove the inoculum. Then they were washed with double-distilled water for 3 times to avoid residual inoculum, finally they were prepared for RNA extraction. The relative quantity of RSV particles RNA was determined using RT-qPCR and DIG-northern blot.

### RNA interference

A partial sequence of LsST6 and GFP was amplified using specifically designed primers as templates to synthesize dsRNA using the protocol for the T7 RiboMAX Express RNAi System. Approximately 400 third-instar and nonviruliferous nymphs of *L*. *striatellus* were injected with 23 nl dsLsST6 (2.5 μg/μl) or GFP (2.5 μg/μl) using an Auto-Nanoliter Injector (Drummond, USA) and then allowed to feed on the RSV-infected rice plant for a 2-day acquisition access period (AAP). At 2, 4 and 8 days after the AAP, RNA was extracted from 50 insects to quantify the LsST6 and RSV mRNA levels by RT-qPCR and DIG-northern blot, and the midgut tissues were excised from 50 insects for immunofluorescence assay. The remaining insects were tested for RSV by RT-PCR with RSV-specific primers. To identify the influence of injection on RSV titre and transmission in insects, we injected 400 third-instar and viruliferous nymphs with 23 nl dsLsST6 (2.5 μg/μl) or dsGFP (2.5 μg/μl) and then transferred them to healthy rice seedlings. Fifty insects were collected for RNA extraction and detection at 2, 4 and 8 days after injection. One hundred insects were transferred to healthy seedlings (1/seedling) for a 10 h inoculation access period, and the seedlings were then grown in a greenhouse for 3 weeks. The infection status of each seedling was assessed by RT-PCR using specific primers for RSV NP. Midguts of the remaining insects were excised for immunofluorescence assay. RT-qPCR and northern blot were used to quantify any changes in RNA levels for RSV and LsST6, and LSCM was used to visualize RSV particles in the excised midgut of *L*. *striatellus*. Each set of experiments was repeated three times.

### RT-qPCR

cDNA was synthesized from 1 μg of total RNA using a FastQuant RT Kit according to the manufacturer’s instructions at 4°C for 3 min, 42°C for 15 min, and 95°C for 3 min. The RT-qPCR was performed using a SuperRealPreMix Plus (SYBR Green) kit, a reaction volume of 20 μl (10 μl of PCR buffer, 0.6 μl of each primer [10 μM/μl], 3 μl of template cDNA, and 5.4 μl of DEPC H_2_O and 0.4 μl 50× ROX Reference Dye) and ABI-7500 thermocycler (Applied Biosystems). The thermocyling program was 94°C for 15 min, followed by 40 cycles of 95°C for 10 s and 60°C for 32 s. Fluorescence was measured at the end of every 60°C extension phase. Beta-actin of SBPHs or ecdysoneless (ECD) of Sf9 cells were used for normalization as housekeeping genes in respective experiments. RT-qPCR data were analysed using the Livak method (2^−ΔΔCt^) [63]. The experiments were repeated 3 times independently.

### DIG-northern blot assay

RNA probes for LsST6 and RSV detection were labeled with DIG using the DIG Northern Starter Kit according to the manufacturer’s instructions. Total RNA extracted from SBPH or Sf9 cells was separated in 1.2% formaldehyde agarose gel and then transferred to nylon membranes using a vacuum regulator (Bio-Rad, USA) for 3–4 h. The membranes were then incubated with the respective RNA probes for 16–20 h in a 65°C hybridization oven, incubated with anti-digoxigenin-AP for 30 min, then in CDP-Star solution for 5 min and imaged with the imageQuant LAS 4000 mini (GE, USA).

### Immunofluorescence microscopy

Sf9 cells previously fixed on cover slips were incubated in 4% (wt/vol) paraformaldehyde for 30 min at room temperature. After being washed 3 times with PBS buffer, the samples were then incubated in osmotic buffer (2% [vol/vol] TritonX-100 in PBS) for 15–30 min at room temperature, then incubated with anti-Myc monoclonal antibody (MAB) (1:400), anti-His MAB (1:400), anti-His rabbit polyclonal AB (1:400) or anti-RSV MAB (1:500) in PBS containing 3% (wt/vol) BSA at room temperature for 1 h and then with goat anti-mouse (1:400) or goat anti-rabbit (1:400) secondary antibody labeled with Dylight 488 or Dylight 549 in PBS for 1 h at 37°C after extensive washing with PBS buffer. The nucleus was stained with 50 nM 4′,6-diamidino-2-phenylindole (DAPI) in PBS at 37°C for 10 min. Midgut tissues excised from the planthoppers were fixed in 4% (vol/vol) paraformaldehyde for 2 h at room temperature and incubated in osmotic buffer for 30 min at room temperature. Then the samples were incubated in anti-RSV MAB labeled with Dylight 549 (red) or anti-ST6 labeled with Dylight 488 (green) MAB for 1.5 h at room temperature. All samples were visualized with LSCM (Zeiss LSM880, GER), and the images saved in ZEN 2011 blue light.

### Immunoelectron microscopy

Dissected midguts or Sf9 cells were fixed for 2 h in 2% (vol/vol) paraformaldehyde and 2% (wt/vol) osmium tetroxide in PBS, and after sequential dehydration in 30%, 50%, 70%, 90%, 100% and 100% alcohol, midguts or Sf9 cells were embedded in LR Gold Resin (cat. 62659, Sigma). Sections (70–90 nm) of the midguts or Sf9 cells were cut using an ultramicrotome (Leica, GER), then blocked for 30 min in blocking buffer. The sections were then incubated at room temperature with the antibodies in the following order: anti-RSV rabbit serum (1:300) for 1.5 h, 10-nm gold-conjugated goat-anti-rabbit IgG for 1 h, anti-LsST6 mouse serum (1:50) for 1.5 h and 5-nm gold-conjugated goat-anti-mouse IgG for 1 h with a wash in distilled water after each antibody incubation. They were then stained in 2% neutral uranyl acetate (w/v in distilled water) for 10 min. The sections were viewed with a transmission electron microscope at 80 kV accelerating voltage.

### Statistical analysis

Means ± SEM of three independent experiments (one-way ANOVA, least significant difference test) were statistically analysed using Prism 6 software (GraphPad); *P < 0.01 was considered statistically significant.

## Supporting information

S1 Fig Genomic structure of *LsST6*. (A) *LsST6* consisted of 5′UTR (blue), ORF (green) and 3′UTR (yellow). (B) Base sequence of *LsST6* had high identity with *NlST6* from the brown planthopper (*Nilaparvata lugens*). Sequences of *LsST6* and *NlST6* were aligned using ClustalX2 software.
(TIF)

S2 Fig Deduced amino acid sequence and structural domains of LsST6. (A) Alignment of amino acid sequences of LsST6 and NlST6. LsST6 had 91.41% identity with NlST6; 12 predicted transmembrane peptides are boxed in red; a conserved GRK domain is boxed in green. (B) Schematic representation of LsST6. A predicted glycosylation site and two predicted substrate-binding domains are marked. (C) Prediction of signal peptide of LsST6. There was no signal peptide in LsST6 according to the analysis using SignalP4.1 (http://www.cbs.dtu.dk/services/SignalP/).
(TIF)

S3 Fig Distribution of RSV in gut of *L. striatellus*. (A) Alimentary canal of *L*. *striatellus*, comprising the esophagus (es), anterior diverticulum (ad), midgut (mg), hindgut (hg) and malpighian tubules (mts). Dylight 633 phalloidin was used to label actin (blue) in the midgut epithelial cells. Scale bars, 200 μm. (B) RSV spreads in the gut of *L*. *striatellus* after feeding on RSV-infected seedlings for a 2-day acquisition access period (AAP). The gut was excised at different days after AAP and incubated with anti-RSV antibody, then observed with LSCM. Scale bars, 200 μm.
(TIF)

S1 Table Primers and probes used in this study. (TIF)

S2 Table The sequence of *LsST6*. (TIF)

S3 Table RSV acquisition percentage and transmission efficiency of healthy insects of *L. striatellus* injected with dsLsST6 or dsGFP. (TIF)

S4 Table Transmission efficiency of viruliferous insects of *L. striatellus* injected with dsLsST6 or dsGFP. (TIF)
