# Supplementary figures and images for: Invasion of midgut epithelial cells by a persistently transmitted virus is mediated by sugar transporter 6 in its insect vector
Source: PLoS Pathog. 2018 Jul 27;14(7):e1007201. doi: 10.1371/journal.ppat.1007201 (PMC6082570; doi:10.1371/journal.ppat.1007201)

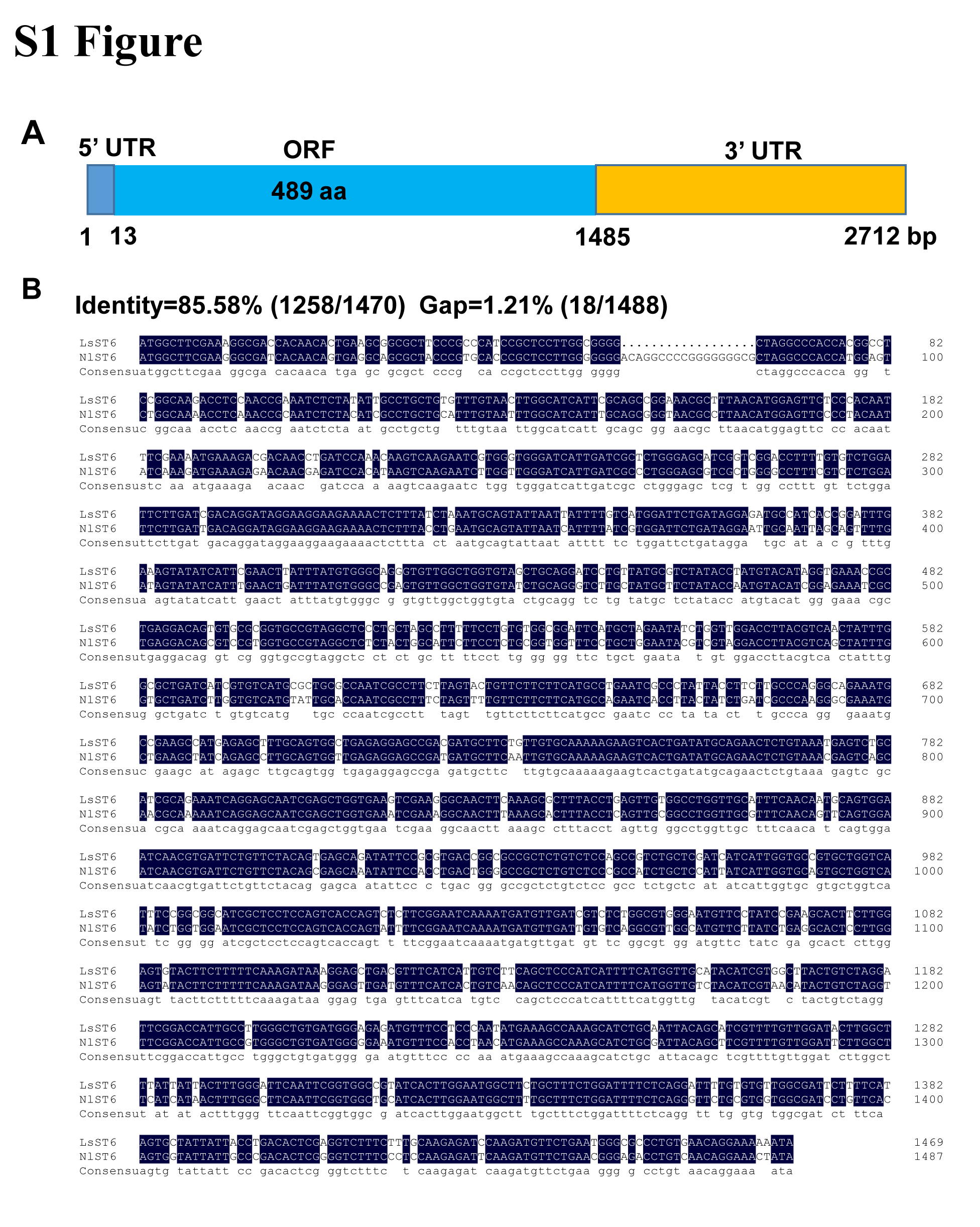

Supplement: S1 Fig — (A) LsST6 consisted of 5′UTR (blue), ORF (green) and 3′UTR (yellow). (B) Base sequence of LsST6 had high identity with NlST6 from the brown planthopper (Nilaparvata lugens). Sequences of LsST6 and NlST6 were aligned using ClustalX2 software. (TIF) [file ppat.1007201.s001.tif]

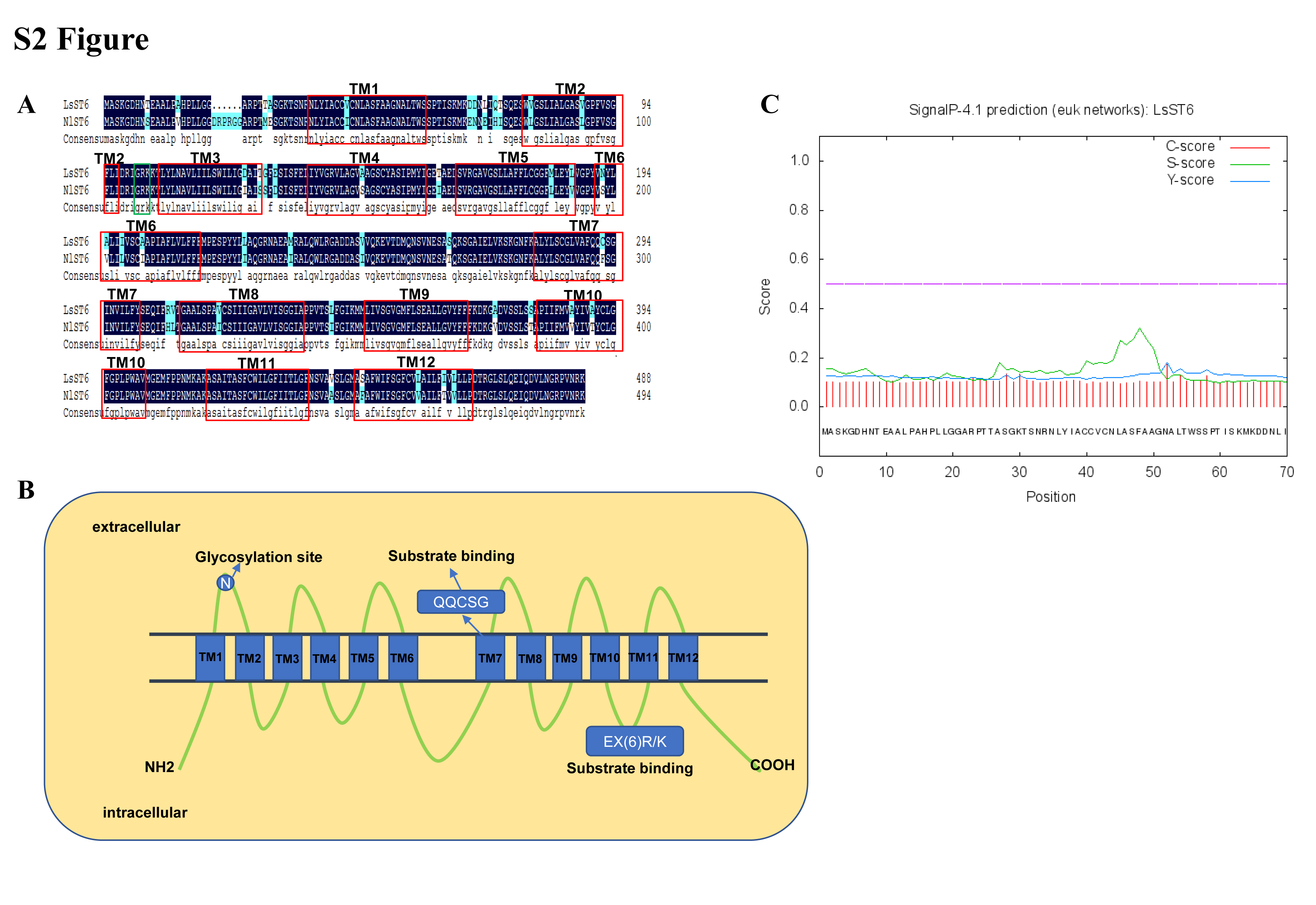

Supplement: S2 Fig — (A) Alignment of amino acid sequences of LsST6 and NlST6. LsST6 had 91.41% identity with NlST6; 12 predicted transmembrane peptides are boxed in red; a conserved GRK domain is boxed in green. (B) Schematic representation of LsST6. A predicted glycosylation site and two predicted substrate-binding domains are marked. (C) Prediction of signal peptide of LsST6. There was no signal peptide in LsST6 according to the analysis using SignalP4.1 (http://www.cbs.dtu.dk/services/SignalP/). (TIF) [file ppat.1007201.s002.tif]

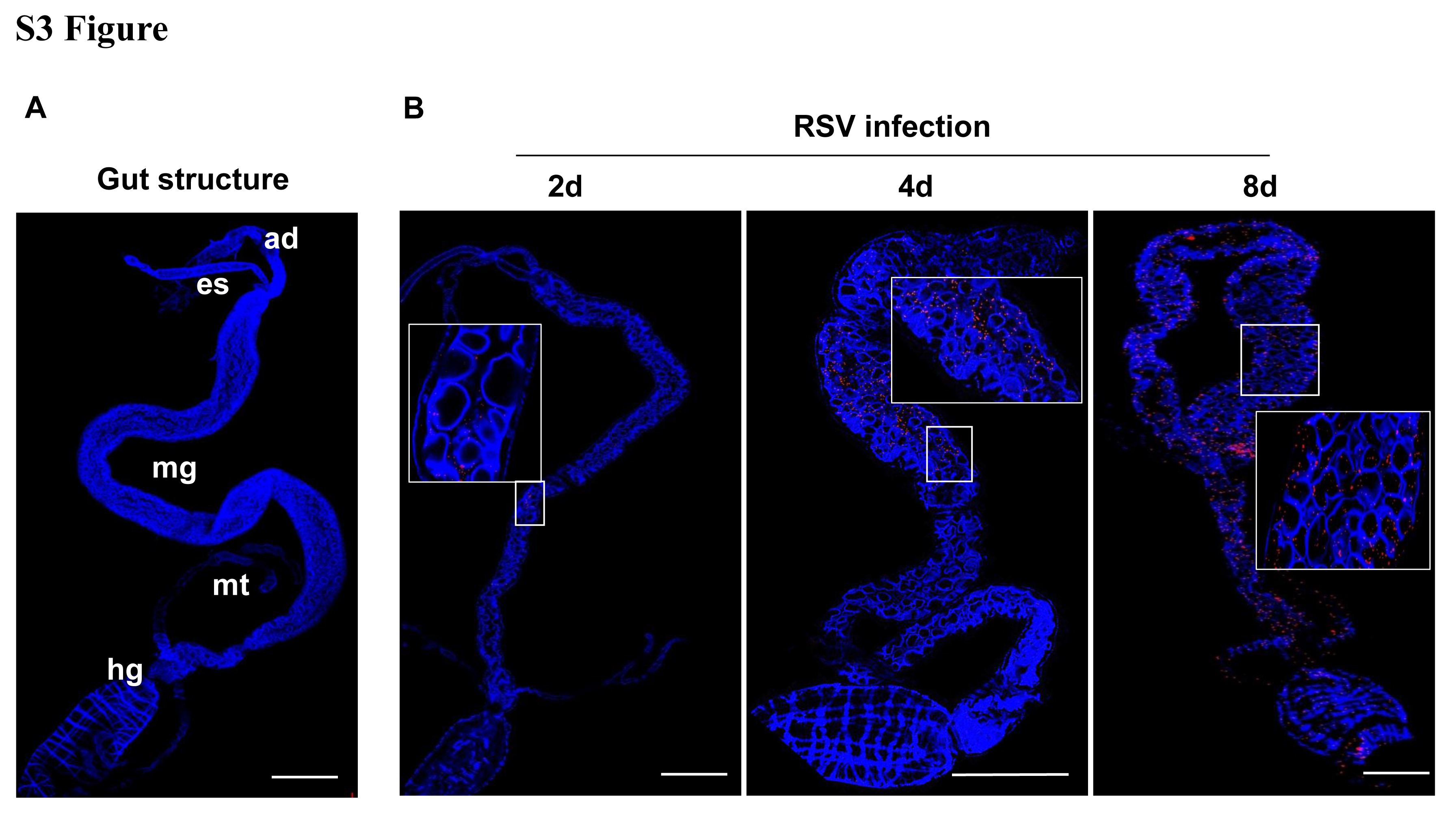

Supplement: S3 Fig — (A) Alimentary canal of L. striatellus, comprising the esophagus (es), anterior diverticulum (ad), midgut (mg), hindgut (hg) and malpighian tubules (mts). Dylight 633 phalloidin was used to label actin (blue) in the midgut epithelial cells. Scale bars, 200 μm. (B) RSV spreads in the gut of L. striatellus after feeding on RSV-infected seedlings for a 2-day acquisition access period (AAP). The gut was excised at different days after AAP and incubated with anti-RSV antibody, then observed with LSCM. Scale bars, 200 μm. (TIF) [file ppat.1007201.s003.tif]

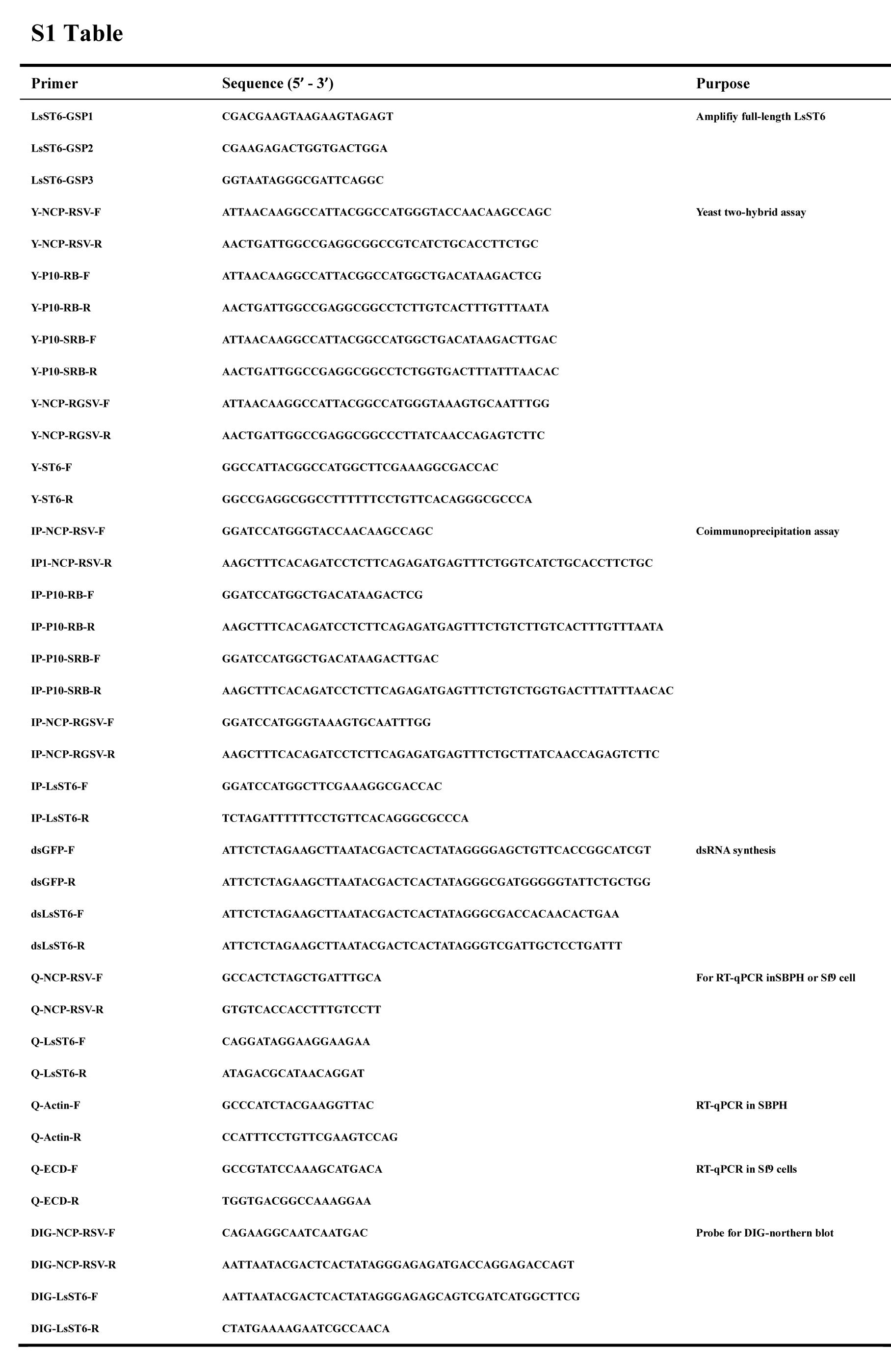

Supplement: S1 Table — (TIF) [file ppat.1007201.s004.tif]

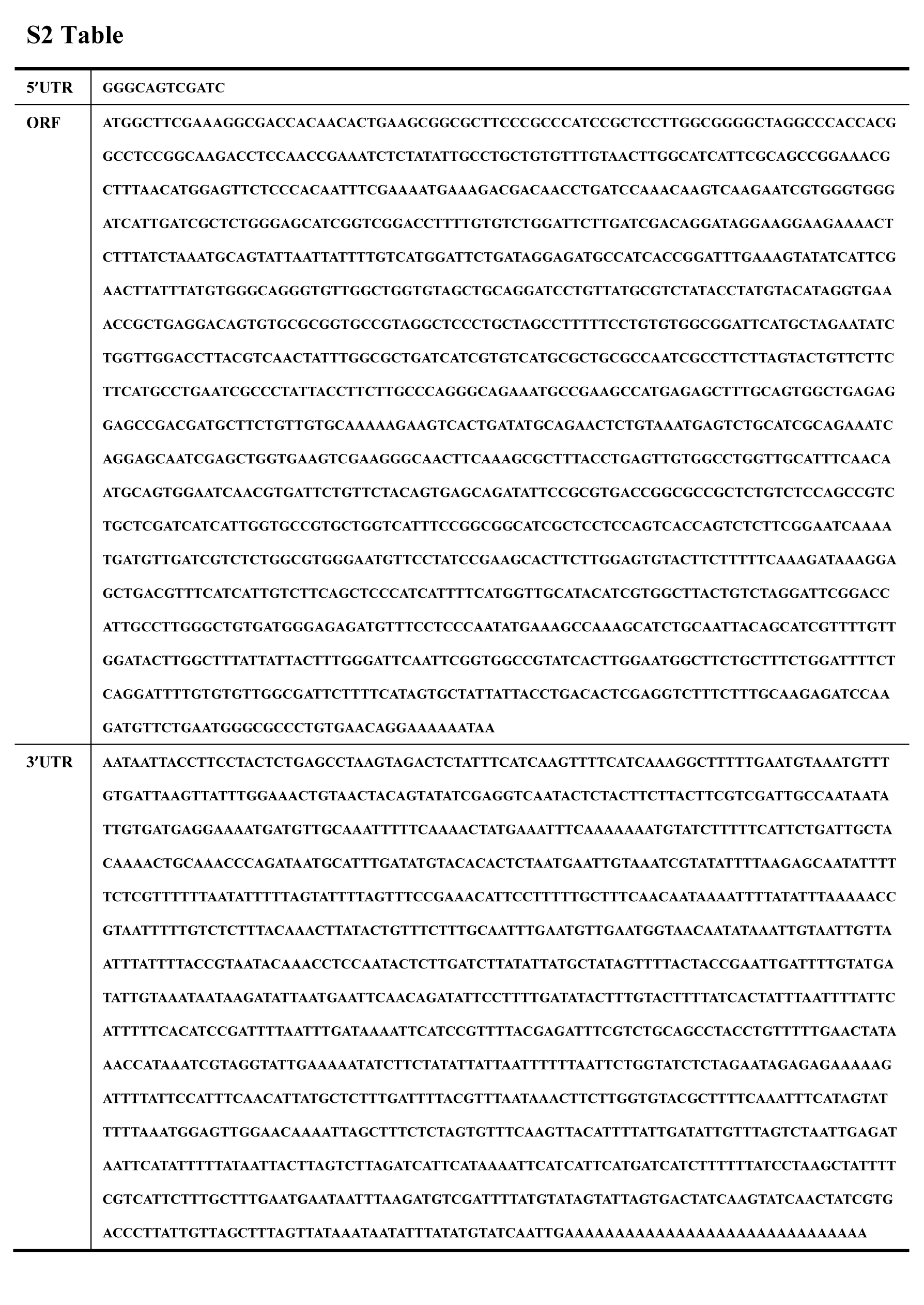

Supplement: S2 Table — (TIF) [file ppat.1007201.s005.tif]

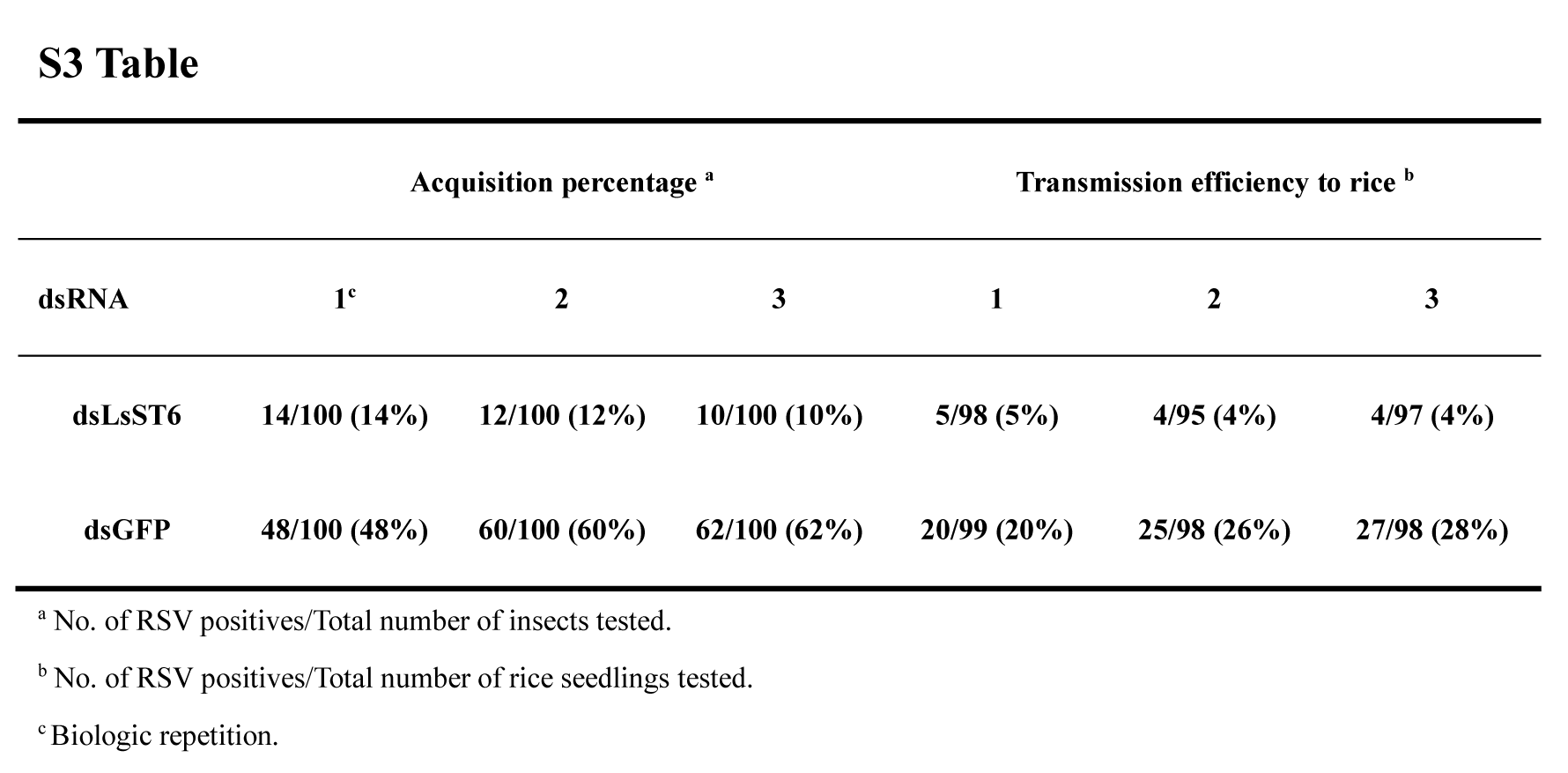

Supplement: S3 Table — (TIF) [file ppat.1007201.s006.tif]

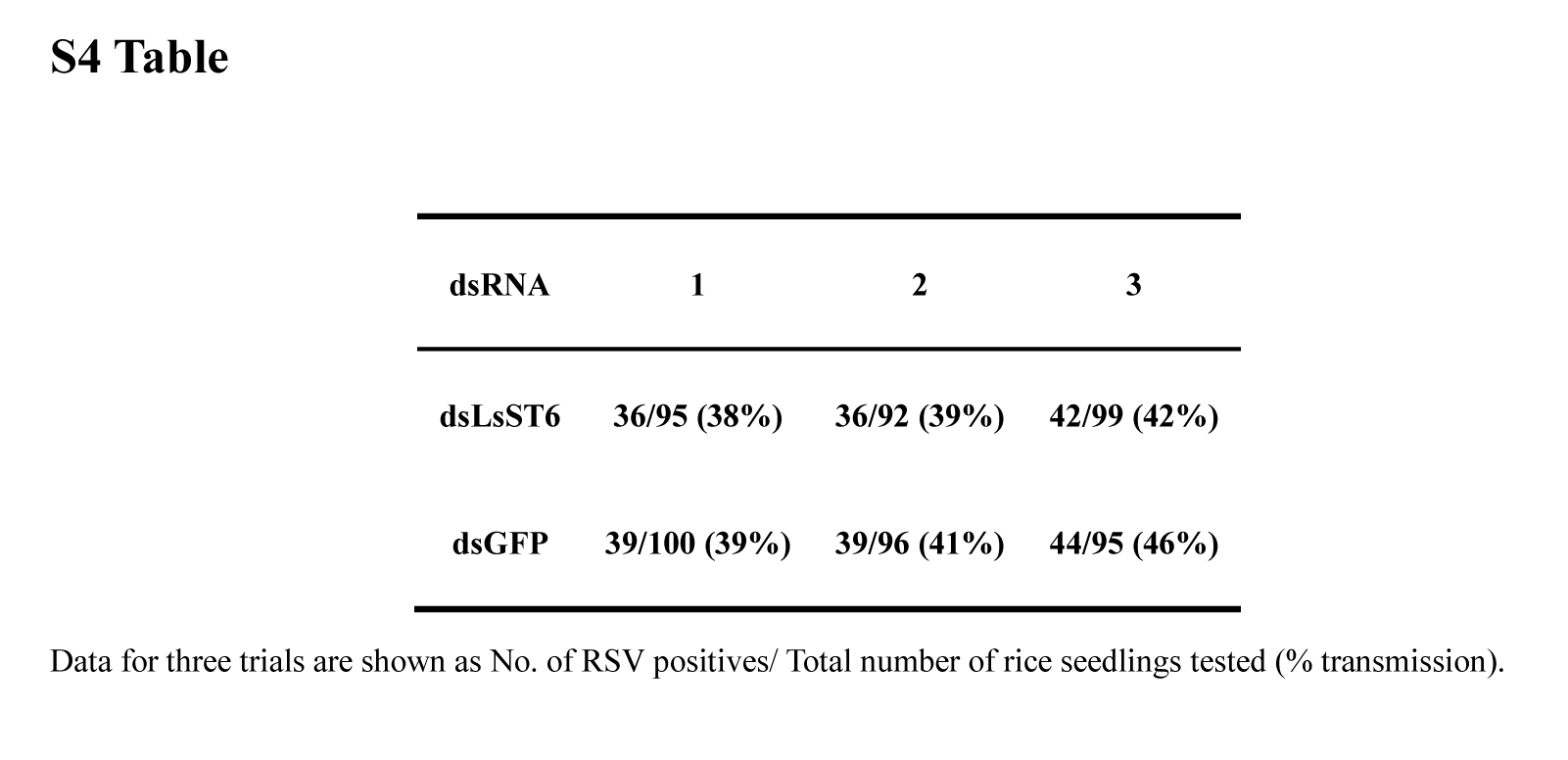

Supplement: S4 Table — (TIF) [file ppat.1007201.s007.tif]
